# 

**Published:** 1916-11

**Authors:** 


### NEW SUBSCRIBERS

Drs. Hoover & Hoover -- Ky.
Dr. Geo. M. Royster ----- Ky.
Dr. Wm. H. Wilson ----- Ky.
Dr. W. T. Travis -------- Ky.
Dr. C. E. Feddeman ----- Va.
Dr. R. A. Barnett ----------Fla.
Dr. S. R. Horton ---------N. C.
Dr. L. M. Nance ----------Texas
Dr. Morris Henry ----------Ark.
Dr. E. Depew ------------Texas
Dr. J. W. Cox ------------Texas
Dr. S. P. McConnell --------Ark.
Dr. T. R. Welch -------------Ky.
Dr. C. W. Bonner ----------N. C.
Dr. O. D. Baxter ----------N. C.
Dr. L. W. lias ---E-------N. C.
Dr. B. W. Fassett --------N. C.
Natchez Hospital --6-------Miss.
Dr. J. S. Slicer -------------Va.
Dr. A. L. Evans ------------Fla.
Dr. W. L. Dunn -----------N. C.
Dr. C. J. Carmichael ------Tenn.
Dr. J. L. Taylor ---------Texas
Dr. D. L. Walker ---------Texas
Dr. D. L. Walker -----------Ky.
Dr. L. S. Booker ----------N. C.
Dr. E. R. Lenep ----------Tenn.
Dr. Frank W. Haines -------Val
Dr. J. G. Flynn -----------Texas
Dr. J. W. Horton --------Tenn.
Dr. B. F. Smith, Jr. ------Texas
Dr. H. H. Holiman ---------Ga.
Dr. W. H. Evans ------- Va.
Dr. B. Kinsell -------- Tex.
Dr. T. J. Crowe -------- Tex.
Dr. H. C. Sauls ---------- Ga.
Dr. O. D. Baxter ------ N. C.
Dr. Wm. T. Smith -----------Ga.
Dr. E. M. Armstrong ------Texas
Dr. J. B. Huff -----------Texas
Dr. J. J. Barton ------------Ga.
Dr. J. H. Downy -----------Ga.
Dr. H. W. Higginbotham ----Ark.
Dr. R. M. Bobbett ------W. Va.
Dr. H. T. Coulter ---------Texas
Dr. A. R. Nicholson --------S. C.
Dr. C. J. Lewis ------------Fla.
Mr. M. L. Murphy ----------Ky.
Dr. A. F. Higgins ----------Fla.
Dr. W. W. Tompkins ----W. Va.
Mr. M. M. Saliba ---------N. C.
Dr. Albert O. Parrott -----N. C.
Dr. J. E. Johnson -----------Ga.
Dr. A. M. Stamps --------Texas
Dr. W. M. Wolfe --------Texas
Dr. W. E. Ramsey --------Texas
Dr. E. C. Tariafero ---------Va.
Dr. H. M. Branham --------Ga.
Dr. W. T. Rowland ---------Ark.
Dr. E. W. Young ------------Va.
Dr. J. E. Cannady -------W. Va.
Dr. H. L. Leathers ----------Ark.
Dr. Kotz. Allen -----------Miss.
Dr. G. C. Schoolfield -----W. Va.

Dr. M. H. Cobb ___________Ga.
Dr. J. W. Pottenhaue ______Texas
Dr. J. P. Oldham _________Texas
Dr. W. R. Potter _________Texas
Dr. A. M. Showarter ________Va.
Dr E. W. Horton ________W. Va.
Dr. C. C. Prichard _______W. Va.
Dr. S. S. Martin __________Texas
Dr. W. J. Davidson ______W. Va.
Dr. A. D. Simington ________Ala.
Dr. W. Morrow ____________Tex.
Dr. W. Gammon ____________Tex.
Dr. E. F. Brewer ___________Ark.
Dr. Albert Green ___________Tex.
Dr. J. L. Lewis ____________Okla.
Dr. S. T. Christopher ________Va.
Dr. J. B. Thielen __________Tenn.
Dr. F. B. Morgan _________Texas
Dr. C. S. Lawrence ________N. C.
Dr. W. D. Holloway ______Tenn.
Dr. G. A. Cooley __________Texas
Dr. L. Kirby ______________Ark.
Drs. Fox & St. Clair______W. Va.
Dr. C. E. WCoxton ________N. C.
Dr. Wm. Breathwit _________Ark.
Dr. M. P. Wilson _________Texas
Dr. H. G. Stoneham _________Va.
Dr. C. W. Doughtie __________Va.
Drs. Wilis,, Knighton, Nelson__La.
Dr. C. M. Truschell ______W. Va.
Dr. Jno. W. Kinnis ________Tex.
Dr. W. H. L. Whitel ______Tenn.
Dr. C. T. Taylor ________W. Va.
Dr. D. C. Mayes ________W. Va.
Dr. T. W Carroll _________Texas
Dr. A. M. Durham _________Ga.
Dr. J. T. Lawson ________Texas
Dr. D. W. Turner _________Miss.
Dr. Jas. Greenwood ______Texas
Dr. Dick P. Wall _________Texas
Dr. Wm. F. Stone ________Tenn.
Dr. Albert G. Kern _______Tenn.
Dr. T. W. Robertson _____Texas
Dr. J. S. Daniel ____________Ga.
Dr. C. D. Baird __________N. C.
Dr. Jas. F. Hicks ________W. Va.
Dr. H. D. Hatfield ______W. Va.
Drs. Woodon & Hall ________Ga.
Dr. J. C. Michael ________Texas
Dr. A. B. Small __________Texas
Dr. E. W. Grey ____________Ga.
Dr. Anderson Watkins ____Ark.
Dr. J. P. Bates ___________Miss.
Dr. M. L. Richardson ______Va.
Dr. H. L. Stinson __________Mo.
Dr. E. H. Cary ___________Texas

Drs. Newell & Newell ____Tenn.
Dr. Curry Wagoner ______Texas
Dr. R. T. Morris _________Texas
Dr. J. C. Ralston ________Texas
Dr. R. V. Brawley _______N. C.
Dr. W. B. Russ __________Texas
Dr. L. L. Blecher ______W. Va.
Dr. D. M. Kipps __________Va.
Dr. Geo. H. Lee__________Texas
Dr. F. L. Paschal ________Texas
David P. Houston _______Tenn.
M. E. Gilmore ____________Texas
Dr. J. D. Donald __________Miss.
Dr. E. R. Cothan __________Ark.
Dr. J. Scarborough ________Ark.
Dr. S. H. McLean ________Miss.
Dr. S. D. Bevill ___________Okla.
Dr. E. M. Perdue ___________Mo.
Dr. J. W. Evans ________Texas
Dr. H. H. McCampbell ____Tenn.
Dr. W. H. Taylor ________Tenn.
Dr. P. H. Stewart __________Ky.
Dr. F. B. F. Hardison _____S. C.
Norfolk Co. Med. Society____Va.
Dr. A. T. Prichard ________N. C.
Dr. Philip C. Jackson______N. C.
Dr. J. L. Rawls _____________Va.
Drs. Dicky & Dicky________Texas
Dr. R. P. Spurlin __________Ark.
Dr. Chas. C. Blake _________Fla.
Dr. C. S. Dodd ______________Va.
Dr. C. A. Woodard ________N. C.
Dr. E. H. Johnson _________Ark.
Dr. J. B. Shelmire ________Texas
Dr. T. J. Charlton __________Ga.
Dr. S. N. Harrell _________N. C.
Dr. V. D. Howard ________Tenn.
Dr. M. E. Leland ___________Ky.
Dr. W. J. Masters _______Texas
Dr. O. P. Schaub _________N. C.
Dr. Joseph Leland __________Ala.
Dr. W. P. Doyle ____________Va.
Dr. J. S. McKenzie _________Ga.
Dr. W. J. McAnnaly _______N. C.
Dr. N. E. Benson ___________Ga.
Dr. Addison Brenizer _____N. C.
Dr. Wm. Bowen __________Tenn.
Dr. A. P. Latham ___________Ky.
Dr. C. W. Rain ____________Tenn.
Dr. Chas. S. Jordan _______N. C.
Dr. W. R. Brandon _______N. C.
Dr. B. J. Witherspoon _____N. C.
Dr. H. C. McCullough ______Ala.
Dr. O. L. Williamson _______Ark.
Dr. J. C. Watkins _________N. C.
Dr. C. S. Rockwell __________Ga.

